# Exploring the relationships between International Classification of Functioning, Disability and Health (ICF) constructs of Impairment, Activity Limitation and Participation Restriction in people with osteoarthritis prior to joint replacement

**DOI:** 10.1186/1471-2474-12-97

**Published:** 2011-05-16

**Authors:** Beth Pollard, Marie Johnston, Paul Dieppe

**Affiliations:** 1 Aberdeen Health Psychology Group, University of Aberdeen, Aberdeen, UK; 2 Peninsula Medical School, University of Plymouth, Plymouth, UK

## Abstract

**Background:**

The International Classification of Functioning, Disability and Health (ICF) proposes three main constructs, impairment (I), activity limitation (A) and participation restriction (P). The ICF model allows for all paths between the constructs to be explored, with significant paths likely to vary for different conditions. The relationships between I, A and P have been explored in some conditions but not previously in people with osteoarthritis prior to joint replacement. The aim of this paper is to examine these relationships using separate measures of each construct and structural equation modelling.

**Methods:**

A geographical cohort of 413 patients with osteoarthritis about to undergo hip and knee joint replacement completed the Aberdeen measures of Impairment, Activity Limitation and Participation Restriction (Ab-IAP). Confirmatory factor analysis was used to test the three factor (I, A, P) measurement model. Structural equation modelling was used to explore the I, A and P pathways in the ICF model.

**Results:**

There was support from confirmatory factor analysis for the three factor I, A, P measurement model. The structural equation model had good fit [S-B Chi-square = 439.45, df = 149, CFI robust = 0.91, RMSEA robust = 0.07] and indicated significant pathways between I and A (standardised coefficient = 0.76 p < 0.0001) and between A and P (standardised coefficient = 0.75 p < 0.0001). However, the path between I and P was not significant (standardised coefficient = 0.01).

**Conclusion:**

The significant pathways suggest that treatments and interventions aimed at reducing impairment, such as joint replacement, may only affect P indirectly, through A, however, longitudinal data would be needed to establish this.

## Background

Osteoarthritis (OA) in the lower limbs (hips and knees) is one of the commonest cause of physical disability in older people [1]. Many treatments are available, some of which target an impairment, such as pain, or restrictions of joint movement, and some on activities limitations, such as reduced walking ability or difficulties with stair climbing (e.g.[2-5]). However, an important issue, for people with OA is to improve their participation in society which has been restricted by the impairments and activities limitations [6-8]. It is therefore important to know the relationships between impairments, activities limitations and restricted participation in this patient group.

The leading model of disability is the International Classification of Functioning, Disability and Health (ICF) [9]. The ICF proposes three main constructs, impairment (I), activity limitation (A) and participation restriction (P) together with contextual factors (personal and environmental factors). The ICF model appears to be a blank canvas waiting for parameters to be identified for various conditions. For example, it has been suggested that for vitiligo the expected significant pathways may be between I and P without an impact on A, whereas, for leprosy, the expected significant pathways may be between all three constructs [10].

**Figure 1:**
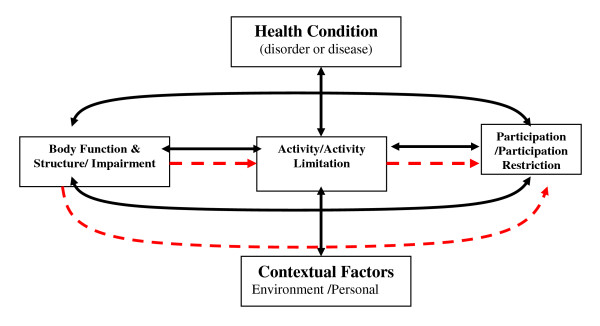
**The ICF model (with basic pathways indicated by dashed line)**.

The model can be complex with feedback loops between the constructs and with the inclusion of potentially important contextual factors. However, in the first instance, there is value in exploring the basic, simple relationships between I, A and P (i.e. the paths from I to A, A to P and I to P) rather than exploring more complex models (see Figure 1).

As yet, only a few studies have empirically explored the relationships between the constructs in the ICF model by condition (e.g. AIDS [11] and Leprosy [12] and distal radius fracture [13]). Within the arthritic conditions, relationships for all or a part of the ICF model have been explored for ankylosing spondylitis, juvenile idiopathic arthritis, rheumatoid arthritis and osteoarthritis. Within juvenile idiopathic arthritis strong relationships were found between I, A and P [14]. Significant relationships between I, A and P were also found in patients with ankylosing spondylitis, however A and P were only partly explained by I [15].

The basic pathways of the ICF have been explored in people with rheumatoid arthritis. Good support was found for the relationship between I and A, and A and P, but less evidence of the direct path between I and P with only 1 of 12 correlations being substantial [16]. Kuhlow et al [17] also found significant associations, in patients with RA, between I and A with A mainly explained by vitality (I) and disease but P was mainly explained by vitality and mental health, both I components.

In a community sample of people with OA the relationship between physical symptoms (I) and participation restrictions was found to be mediated by activity limitations and depression [18]. For patients with hand osteoarthritis: hand related activity was related to impairment; but when a general measure of A and P was used, A and P were more strongly related to personal factors than I. [19]. This illustrates the importance of the content of measures. One pathway between I and A has been explored in patients prior to joint replacement as part of the exploration of integrated ICF and psychological models [20]. Evidence of a significant pathway between I to A was found, but pathways involving P were not explored. Here, we extend the exploration of the model for OA by exploring the basic pathways (i.e. including the paths between I and P and A and P).

It is important to ensure that measures assess only the construct of interest and are not simultaneously measuring other constructs within the model or outwith the model. If measures are not 'uncontaminated' (i.e. only measuring the construct of interest), empirical evidence for relationships between constructs in the model may be misleading. To address this issue, in this study we use specifically developed statistically separable measures of I, A and P. We used structural equation modelling (SEM) as these methods can evaluate models of both the measurement of the constructs and the structure of the relationships between constructs.

Hence, the aim of this study was to explore the basic ICF pathways (i.e. from I to A, A to P and I to P) for people with OA prior to joint replacement surgery using structural equation modelling.

## Method

### Design

A geographical cohort of patients with osteoarthritis from the Tayside Joint Replacement (TJR) cohort about to undergo hip or knee joint replacement surgery at Ninewells Hospital, Dundee, Scotland, completed measures of I, A and P. Structural equation modelling (SEM) was used to explore the relationships between the constructs.

### Participants

The study sample comprised 413 people having their first hip or first knee replacement (on average 34 days before surgery). All of the sample had received a confirmed diagnosis of OA from a consultant orthopaedic surgeon. A subset of these patients was included in the sample previously investigated by Dixon et al [20].

A written informed consent was obtained from all patients. Ethics approval was obtained from the Tayside Committee on Medical Research Ethics and was conducted in accordance with the Helsinki Declaration.

### Measures

The main constructs of the ICF model were measured using the Ab-IAP (the Aberdeen measures of Impairment (Ab-I), Activity Limitation (Ab-A) and Participation Restriction (Ab-P) [21]. The Ab-IAP was developed specifically to measure the ICF constructs of impairment, activity limitation and participation restriction. The Ab-IAP items were selected from a pool of items from common osteoarthritis measures that were classified as measuring only I or A or P by expert judges using discriminant content validity [22]. The measures were established using a combination of confirmatory factor analysis (CFA), item response theory (IRT) and classical test theory (CTT) to eliminate items from the Ab-IAP which performed poorly. Before examining the pathways it was necessary to establish statistically separable measures of the constructs so that the results would not be confounded due to measures being contaminated with other constructs in the model. Details of the development and validation of the separable measures are in Additional file 1. These measures are referred to as the modified Ab-IAP measures and comprise the 7 item Ab-I_(mod)_, the 7 item Ab-A_(mod) _and 5 item Ab-P_(mod). _The items and response categories are shown in Table 1. The American knee score [23] and the Harris hip score [24] were used to compare non-responders to responders. The American knee score measures impairment and activity limitation and the Harris hip score measures impairment, activity limitation with some mixed items [22].

**Table 1:** Items in the modified Ab-I, AB-A and AB-P measures

I items: AB-I _(mod)_	Response categories
I1. How would you describe the pain you usually have from your joint?	None, Mild, Moderate, Severe, Extreme

I2. How often have you had severe pain from your arthritis?	Never, Occasionally, Quite Often, Most of the time, All of the time

I3. Does remaining standing for 30 minutes increase your pain?	Never, Occasionally, Quite Often, Most of the time, All of the time

I4. How active has your arthritis been?	Not at all, Mildly, Moderately, Severely, Extremely

I5. Have you been troubled by pain from your joint in bed at night?	No nights, Occasional nights, Quite often, Most nights, Every night

I6. How long has your morning stiffness usually lasted from the time you wake up?	No morning stiffness, Less than 30 minutes, 30 minutes to 1 hour, 1 to 2 hours, Over 2 hours

I7. How severe is your stiffness after first wakening in the morning?	None, Mild, Moderate, Severe, Extreme

**A items: AB-A _(mod)_**	

A1. What degree of difficulty do you have rising from sitting?	None, Mild, Moderate, Severe, Extreme

A2. What degree of difficulty do you have rising from bed?	None, Mild, Moderate, Severe, Extreme

A3. What degree of difficulty do you have sitting?	None, Mild, Moderate, Severe, Extreme

A4. What degree of difficulty do you have getting on/off toilet?	None, Mild, Moderate, Severe, Extreme

A5. What degree of difficulty do you have climbing up and down one flight of stairs?	None, Mild, Moderate, Severe, Extreme

A6. What degree of difficulty do you have dressing yourself (except socks and shoes)?	None, Mild, Moderate, Severe, Extreme

A7. What degree of difficulty do you have washing and drying yourself?	None, Mild, Moderate, Severe, Extreme

**P items: AB-P_(mod)_**	

P1. How does your joint problem restrict you having friends or relatives over to your home?	Not at all, A little, Moderately, Severely, Extremely

P2. How does your joint problem restrict you visiting friends or relatives?	Not at all, A little, Moderately, Severely, Extremely

P3. How does your joint problem restrict you telephoning friends or relatives?	Not at all, A little, Moderately, Severely, Extremely

P4. How does your joint problem restrict you doing your usual social activities?	Not at all, A little, Moderately, Severely, Extremely

P5. How much of the time has your physical health or emotional problems interfered with your social activities (like visiting with friends)	All of the time, Most of the time, Some of the time, A little of the time, None of the time

### Procedure

A questionnaire pack was sent to each patient's home approximately four weeks prior to surgery by the pre-operative assessment nurse at Ninewells Hospital. The questionnaire pack consisted of an invitation to participate, patient information sheet, consent form, questionnaire and stamped return envelope. The patients completed the questionnaire at home and returned it by post to the research team at Ninewells Hospital, Dundee, Scotland prior to admission for surgery.

### Analysis

#### Structural equation modelling

Structural equation modelling (SEM) was used to explore the relationships between the ICF constructs. The first stage of SEM is to establish an acceptable measurement model and then to add directional paths to form the structural model. EQS version 6.1 [25] was used for the analysis.

#### The measurement model

Confirmatory factor analysis was used to assess the measurement model. The three factor model was explored (i.e. with Ab-I_(mod)_, Ab-A_(mod) _and Ab-P_(mod) _items being indicators of three underlying latent constructs). As standard, one indicator factor loading was set to one, and correlations between the underlying latent factors were free to be estimated. As some items did not appear to be normally distributed, robust Maximum Likelihood estimation was used together with robust fit statistics and robust standard errors. Satorra and Bentler [26] have developed robust statistics, for confirmatory factor analysis, that can take into account departures from non-normality. Hence, where possible robust statistics were used.

The Satorra-Bentler Chi-squared statistic [26] was calculated to assess model fit. As it has been shown that with large samples Chi-square based statistics are often highly significant even if there is good model fit [25], other fit indices were also explored. Model fit was assessed with emphasis on the robust comparative fit index (CFI), and the robust Root Mean Squared Error of Approximation (RMSEA) with the 90% confidence interval. A CFI>0.90 has been considered satisfactory for model fit [27,28]. A RMSEA value of <= 0.08 is generally accepted as an upper bound for acceptable fit [29].

#### The structural model: Exploring the relationships between I, A and P

Once the measurement model was established then the relationships between the ICF constructs were explored. SEM allows for the estimation of paths where directional relationships between latent constructs have been hypothesised. The final measurement model was used to explore the structural paths using structural equation modelling (SEM). The correlations between the constructs were replaced by directional paths to explore the basic pathways (i.e. paths from I to A, A to P and I to P). Robust statistics were used to test the significance of the path coefficients.

There is not a consensus on sample size for SEM. A minimum sample of 200 has been recommended (e.g.[30]) however some authors suggest 400-500 participants are needed (e.g. [31]). Another method of determining adequate sample size is to have 10 participants per free parameter. In this study we estimated 41 parameters and hence had the required number of participants [32,33]. Thus the study should have sufficient power.

## Results

Thirty-two percent of the study sample had their right hip replaced, 26% had their left knee replaced and 23% had their left hip replaced and 19% had their right knee replaced. The response rate was 38% (i.e. 1096 people were sent the questionnaire). Patient demographics and descriptive statistics on the main measures are presented in Table 2. There was no difference between responders and non-responders in the proportion of men to women or on Harris Hip scores but the non-responders were significantly older (mean age 70.9 yrs (s.d 8.64), t(1094) = 3.60 p < 0.0005) and had worse American knee scores (mean score non-responders = 30.24 (s.d. = 13.3), mean score responders = 32.22 (s.d. = 13.2); t(494) = -2.39 p = 0.02)

**Table 2:** Patients Characteristics

Gender (male)	188 (45.5%)
Age (years)	68.94 (9.4)

Marital status (married)	281(68%)

Living arrangements (alone)	105 (25.4%)

Ethnicity (white)	410 (99.3%)

Paid employment (yes)	69 (16.7%)

	

Usual Pain	

None	1 (0.2%)

Mild	7 (1.7%)

Moderate	95 (23.1%)

Severe	258 (62.8%)

Extreme	50 (12.2%)

Problems walking on the flat	

None	9 (2.2%)

Mild	50 (12.2%)

Moderate	203 (49.6%)

Severe	129 (31.5%)

Extreme	18 (4.4%)

How social activities restricted	

Not at all	28 (6.8%)

A little	83 (20.3%)

Moderately	119 (29.1%)

Severely	133 (32.5%)

Extremely	46 (11.2%)

	

Ab-I*	25.46 (4.53)

Ab-A*	19.59 (5.30)

Ab-P^	11.53 (4.23)

Values are mean (s.d.) or n(%)

*Max score = 35

^Max score = 25

### The measurement model

The CFA analysis indicated good item fit for the three factor model with correlated factors [SB Chi (149) = 439.61; CFI robust = 0.91; RMSEA robust = 0.07 (CI 0.06-0.08)]. This three factor model was significantly better than any of the two factor models with correlated factors and significantly and substantially better than the one factor model, none of which had acceptable evidence of model fit (see Additional file 2: The measurement model: CFA). Thus, the measurement model provides support for I, A & P being separate constructs rather than a single general concept. All latent constructs were significantly correlated with each other, with the strongest correlations between I and A (*r *= 0.76) and A and P (*r *= 0.76) and then I and P (*r *= 0.58).

### The structural model

**Figure 2:**
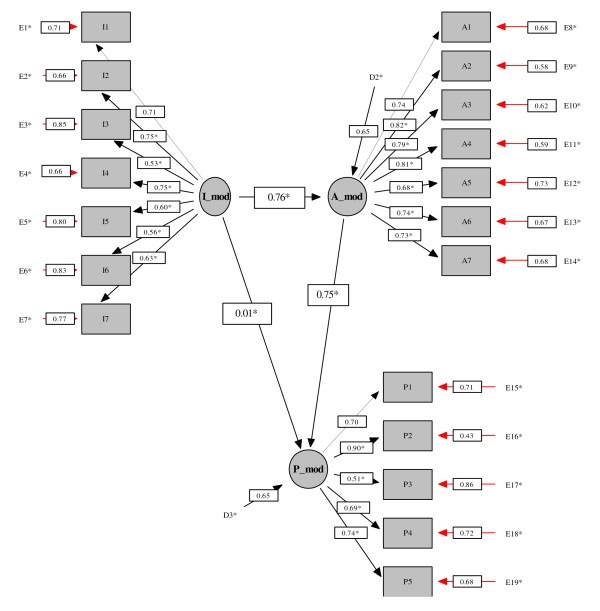
**Structural Equation Model exploring the basic paths of the ICF model for patients with OA prior to joint replacement**. The underlying latent constructs (factors) are represented by the circles. The shaded squares represent the indicators of the underlying construct (i.e. the observed variables). The E's are the error terms of the indicators. The values on the structural paths between the latent constructs are the standardised path coefficients. The values between the latent construct and the indicators are the standardised factor loadings. The D's associated with each latent variable are the residual error in prediction of the unobserved latent construct. The elements marked * are estimated parameters. The path indicated by a dashed line is the path that was set to 1 for identification purposes and so not tested

A structural equation model was fitted to explore the basic ICF pathways (i.e. I to A, A to P and I to P). There was good model fit [S-B Chi-square = 439.45, df = 149, CFI robust = 0.91, RMSEA robust = 0.07; RMSEA robust CI = 0.06-0.08]. All factor loadings were greater than 0.5. There was evidence for significant structural pathways between Ab-I_(mod) _and Ab-A_(mod) _(standardised coefficient = 0.76 p < 0.0001) and between Ab-A_(mod) _and Ab-P_(mod) _(standardised coefficient = 0.75 p < 0.0001). The path between Ab-I_(mod) _and Ab-P_(mod) _was not significant (standardised coefficient = 0.01). The structural pathways with the estimated standardised path coefficients are displayed in Figure 2.

## Discussion

This study explored the basic pathways of the ICF model for people with osteoarthritis prior to joint replacement surgery. Support was found for the ICF pathways between I to A and between A to P, however there was not a significant path between I and P. While this direct I-P path may be relevant for some conditions, it does not appear to be relevant for people with OA. The results are consistent with the findings from a community OA sample where significant paths were found between I and A, and A and P, with activity limitations mediating the relationship between I and P [18]. The results suggest that similar relationships may hold for OA for both severe and milder OA (the community sample had 27% severe pain whereas our sample had 75% severe pain, see Table 2).

Lower limb joint osteoarthritis (OA) is a common cause of disability with the majority of interventions offered to help people with OA, including joint replacement, primarily designed to reduce pain, in the medical belief that pain is the main problem (e.g.[2-5]). However, restricted participation is often an important concern for people with this condition [6-8]. Our results suggest that surgical and pharmacological treatments that improve participation may do so through reducing activity limitations, thus supporting the use of rehabilitation programmes in parallel. The results are also compatible with the finding that interventions which directly address activity, such as pain management programmes, can reduce participation restrictions (e.g.[34]). Thus if patients put more value on participation than impairment outcomes, there is a wider range of possible therapeutic interventions. These results may go some way to explaining the relatively high rates of dissatisfaction with interventions, including joint replacement [35], and suggests that health care professionals need to assess individuals with OA carefully in order to ascertain what they most need to gain from an intervention, and try to target therapy accordingly.

In this study we measured each of the three main constructs rather than take the approach of the WHO ICF research branch http://www.icf-research-branch.org not to differentiate activity limitation from participation restriction [10,36]. For example, they have combined activity limitation and participation restriction in the core ICF category sets for different conditions including OA (e.g. [37-39]). We have found that analysing the results of trials of interventions separating A and P, may offer further insight to the results obtained [40] as some interventions may have more effect on one health component than on others. If empirically possible, there would appear to be great value in measuring A and P separately for both theoretical and practical reasons. Practically, separate measures enable accurate targeting of interventions and may avoid the masking of true treatment effects. For example, if an intervention improves activity limitation but not participation restriction, then a combined measure assessing both activity limitation and participation restriction may indicate a significant effect but would not inform where the intervention has the greatest impact. Theoretically, unless constructs can be measured separately, they are of little value in the model. In addition, it is possible to develop clear testable hypotheses of factors likely to affect participation and activity separately. For example, the social model of disability suggests that social and environmental factors, such as help available and the presence or absence of physical barriers, directly affect participation [41]. Additionally, we have proposed that activity will be determined by cognitions as well as impairments [42] and found evidence that control beliefs such as self-efficacy, as well as individuals' goals or intentions, are important influences on their activity levels [20,43,44] offering support for integrating behavioural models with the ICF model in predicting activity limitations. It remains to be seen, empirically, how these psychological variables impact on the pathways involving participation restriction.

The study has some limitations. We used cross sectional data, whereas longitudinal data may have allowed us to explore causality. It will be possible to investigate a sequential causal model with feedback 'socio-behavioural' routes (i.e. P to A, A to I and P to I) when follow-up post-surgical data is available for these patients. However even with these investigations of predictions over time, these studies cannot test whether the relationships are truly causal and this will require experimental studies such as those conducted by Fisher & Johnston (1996) [45]. In this study we only explored I, A and P and not the contextual factors. These contextual factors may have important moderating or mediating effects on the relationships between I, A and P. The response rate was only 38% but this may be explained by the length of the questionnaire (29 pages) that may have been too burdensome to some patients. The study patients were younger and had better Harris hip scores than non-responders and so this may have introduced some bias in the results.

The measures of I, A and P were derived from the Ab-IAP. The I measure consists of pain and stiffness items and the inclusion of sleep, fatigue or emotional functions may have given different results. Some of the response options for I were frequency-based whereas the A and P measures have severity based options. It is possible that the different type of response options may have affected the CFA. Cieza et al. [46], in developing core sets for various conditions, differentiate impairment into body structure and body function. This was not done in the current study but might be of additional value in conditions such as OA where there are structural changes. While the items in participation restriction measure mainly reflect social functioning they were the most informative items from an item pool that included other participation areas such as transportation, domestic life, interpersonal interactions and relationships and economic life. However, there was an indication that there is a lack of reliability for those at with low participation restriction and so new items could be added to tap this area of the construct (see Additional file 1 figure S3).

## Conclusions

In summary, the significant ICF pathways for OA prior to joint replacement were found to be between I and A and between A and P, but not between I and P. This suggests that A might fully mediate the relationship between I and P but longitudinal data is needed to explore this further. Additional work is necessary to replicate these findings, to investigate reverse causality as proposed by the ICF framework and to test models including social and behavioural factors.

## Competing interests

The authors declare that they have no competing interests.

## Authors' contributions

BP participated in the conception and design of the study, the analysis and the drafting and revision of the manuscript. MJ participated in the conception and design of the study and the drafting and revision of the manuscript. PD contributed to the interpretation of the data and revision of the manuscript. All authors read and approved the final manuscript.

## Pre-publication history

The pre-publication history for this paper can be accessed here:

http://www.biomedcentral.com/1471-2474/12/97/prepub

## Supplementary Material

Additional file 1 **Deriving statistically separable I, A and P measures from the Ab-IAP**. Details of the derivation and validation of statistically separable I, A and P measures from the Ab-IAP. Click here for file

Additional file 2 **The measurement model: CFA**. Details of the measurement model using CFA including comparisons of the three factor model with alternative one and two factor models. Click here for file
