# Supplementary material for: Exploring the relationships between International Classification of Functioning, Disability and Health (ICF) constructs of Impairment, Activity Limitation and Participation Restriction in people with osteoarthritis prior to joint replacement
Source: BMC Musculoskelet Disord. 2011 May 16;12:97. doi: 10.1186/1471-2474-12-97 (PMC3123258; doi:10.1186/1471-2474-12-97)
Supplement: Additional file 1 — Deriving statistically separable I, A and P measures from the Ab-IAP. Details of the derivation and validation of statistically separable I, A and P measures from the Ab-IAP. [file 1471-2474-12-97-S1.DOC]

**Deriving statistically separable I, A and P measures from the Ab-IAP.**

**Method**

**Participants**

The study sample was a geographical cohort of 482 patients prior to having their first hip or knee replacement on that particular joint at Ninewells Hospital, Dundee. The sample is described elsewhere [1]. Of these 482 patients, 413 patients had received a confirmed diagnosis of OA from a consultant orthopaedic surgeon and these constituted the OA sample as used in the main paper.

**Measures**

The Ab-IAP (the Aberdeen measures of Impairment (Ab-I), Activity Limitation (Ab-A) and Participation Restriction (Ab-P)) was used and had been developed specifically to measure the ICF constructs of impairment, activity limitation and participation restriction [1].

**Analysis**

Using EQS version 6.1 [2], confirmatory factor analysis (CFA) was first used to assess if the Ab-I, Ab-A and Ab-P measures were statistically separable. The three factor solution was initially explored (i.e. with Ab-I, Ab-A and Ab-P items being indicators of three underlying latent constructs). Correlations between the latent constructs were free to be estimated. As standard, one indicator factor loading was set to one. As some items did not appear to be normally distributed, robust Maximum Likelihood estimation was used together with robust fit statistics and robust standard errors. Satorra and Bentler [3] have developed robust statistics, for confirmatory factor analysis, that can take into account departures from non-normality. Hence, where possible robust statistics were used.

The Satorra-Bentler Chi-squared statistic [3] was calculated to assess model fit. As it has been shown that with large samples Chi-square based statistics are often highly significant even if there is good model fit [2], other fit indices were also explored. Model fit was assessed with emphasis on the robust comparative fit index (CFI), and the robust Root Mean Squared Error of Approximation (RMSEA) with the 90% confidence interval. A CFI>0.90 has been considered satisfactory for model fit [4,5]. A RMSEA value of <=0.08 is generally accepted as an upper bound for acceptable fit [6].

If the initial three factor model had poor fit, then each construct was then explored separately. If the single factor models did not provide adequate fit then the items within the Ab-I, Ab-A and Ab-P measures were reduced. Within each of the measures, the five items that had previously been shown to have the highest discrimination and information from an Item Response Theory (IRT) analysis, [1] were selected. These five items were supplemented by other items from each measure (i.e. Ab-I, Ab-A, Ab-P) where necessary, to achieve adequate reliability across the construct using the classical test theory criteria of a Cronbach’s alpha >0.8 and good coverage across the construct based on the IRT analysis reported previously) [1]. Post-hoc modifications suggested by the CFA were not used to improve the model fit as overfitting of the data may occur. In addition, CFA methods are sample dependent whereas IRT methods are sample independent.

Each construct was again explored to establish if the single factor modified models were appropriate. If there was acceptable fit then one (IAP), two (IA,P; AP,I; IP,A) and three (I, A, P) factor modified models were fitted with correlations between the underlying latent factors free to be estimated.

If there was acceptable fit then the properties of the separable measures were explored. The internal reliability was explored using Cronbach’s alpha and the reliability across the construct was explored using Item Response Theory (IRT). For the IRT, Samejima’s graded response model was fitted [7]. Model and item fit was evaluated by exploring the differences between observed and model predicted responses in each category. The total test information function provided a test of the reliability of the measure across the whole of the underlying construct. The validity of the separable measures was explored by correlating the measures with appropriate RAND SF-36 subscales [8].

**Results**

CFA indicated that there was poor fit for the three factor model using all the items and just satisfactory model fit for the Ab-I single construct analysis (see Table 1). To improve the model fit, items were removed from Ab-I, Ab-A and Ab-P based on the item response analysis of these items [1]. The specified reduction criteria resulted in 7 Impairment items (the ‘best’ 5 Ab-I items from IRT plus the two next best Ab-I items to ensure Cronbach’s alpha>0.8), 7 Activity Limitation (the ‘best’ 5 Ab-A items from IRT plus 1 Ab-A item with high discrimination at very low end and 1 item at very high end and 5 Participation Restriction items (the best 5 Ab-P items from IRT). The single factor models for the modified

measures of Impairment, Activity Limitation and Participation gave acceptable fit although the RMSEA were higher than usual criterion of 0.08 (Table 2). These modified measures are referred to as Ab-I_(mod)_, Ab-A_(mod)_ and Ab-P_(mod)_.

**Table 1: CFA models on Ab-IAP**

|  | **SB Chi** | **Df** | **CFI robust** | **RMSEA**  **robust** | **RMSEA**  **robust CI** |
| --- | --- | --- | --- | --- | --- |
| Three factor I-A-P | 2323 | 557 | 0.756 | 0.089 | 0.085-0.092 |
| One factor IAP | 7840 | 560 | 0.665 | 0.104 | 0.10-0.107 |
| **One factor I** | **106.73** | **27** | **0.922** | **0.081** | 0.065-0.097 |
| One factor A | 1033.61 | 119 | 0.78 | 0.13 | 0.125-0.140 |
| One factor P | 337.06 | 27 | 0.77 | 0.158 | 0.143-0.173 |

Key: SB Chi= Satorra-Bentler Chi-squared statistic, df=degrees of freedom, CFI robust= robust comparative fit index, RMSEA robust=robust Root Mean Squared Error of Approximation, CI=Confidence Interval.

Bold=acceptable model fit i.e. robust CFI>0.9 and robust RMSEA<0.08.

The three factor model had good fit (this is reported in the main document: results, measurement model). Hence separable measures were able to be established. The validity of these measures was explored.

**Table 2: Measurement Model: CFA on the separable Ab-I, Ab-A, Ab-P measures**

| **MODEL** | **Chi-square** | **Df** | **p** | **SB**  **Chi-square** | **P** | **CFI**  **robust** | **RMSEA**  **Robust** | **RMSEA**  **robust CI** |
| --- | --- | --- | --- | --- | --- | --- | --- | --- |
|  |  |  |  |  |  |  |  |  |
| **Ab-I_(mod)_** | **72.48** | **14** | **0.00034** | **48.41** | **0.00009** | **0.96** | **0.07** | 0.05-0.10 |
| **Ab-A_(mod)_** | **160.99** | **14** | **<0.000005** | **142.21** | **<0.000005** | **0.92** | 0.14 | 0.12-0.16 |
| **Ab-P_(mod)_** | **32.82** | **5** | **<0.000005** | **31.93** | **0.00001** | **0.96** | 0.11 | 0.07-0.14 |
|  |  |  |  |  |  |  |  |  |

Key: SB Chi= Satorra-Bentler Chi-squared statistic, df=degrees of freedom, CFI robust= robust comparative fit index, RMSEA robust=robust Root Mean Squared Error of Approximation, CI=Confidence Interval.

Bold=acceptable model fit i.e. robust CFI>0.9 and robust RMSEA<0.08.

**Validation of Ab-I_(mod)_, Ab-A_(mod)_,Ab-P_(mod)_**

#### Relationship with existing measures

The correlations of the new separable measures of Ab-I_(mod)_, Ab-A_(mod)_ and Ab-P_(mod)_ with the RAND SF-36 subscales were as hypothesised, with each measure correlating highest with the hypothesised RAND SF-36 subscale (see Table 3). However, it is noted that the correlations of Ab-A_(mod)_ with the RAND SF-36 subscales were all of a similar order.

**Table 3: Correlations of RAND SF-36 subscales with the separable** I, A, P measures

|  | **SF-pain** | **SF-phys** | **SF-soc** |
| --- | --- | --- | --- |
| **Ab-I_(mod)_** | **-.62(**)** | -.54(**) | -.45(**) |
| **Ab-A_(mod)_** | -.59(**) | **-.60(**)** | -.59(**) |
| **Ab-P_(mod)_** | -.55(**) | -.56(**) | **-.83(**)** |

** Correlation is significant at the 0.01 level (2-tailed).

SF-pain=SF-36 pain subscale; SF-phys=SF-36 physical subscale; SF-soc=SF-36 social functioning subscale.

#### Reliability of the separable measures

##### Overall

The three measures had acceptable internal reliability with Cronbach’s alpha for Ab-I_(mod)_ (alpha=0.82), Ab-A_(mod)_ (alpha=0.90) and Ab-P_(mod)_ (alpha=0.83).

##### Reliability across the construct

Samejima’s graded response model was fitted (Samejima, 1969). There was very good model fit for Ab-I_(mod)_ and Ab-A_(mod)_ with all response categories having an observed-expected difference of <0.01. For Ab-P_(mod)_, there was also good model fit with 16 of the 20 response categories having a difference of <0.01 but less than 0.02.

The test information function indicated Ab-I_(mod)_ was reliable across the construct except at very high levels of Impairment (Figure 1). Ab-A_(mod)_ appeared reliable across the construct except at the very low end of Activity Limitation (Figure 2). Ab-P_(mod)_ appeared reliable in the range of construct from -1.5 to 2.5 (Figure 3). However, the measure may have reliability problems at low levels of Participation Restriction (i.e.< -1.5). This can also be seen by examining the distributions of the Ab-P_(mod)_ items. Many items were positively skewed with large numbers in the first frequency category i.e. not at all. New uncontaminated items could be added to cover these areas with low reliability. In general, it appears that the selection method resulted in measures with evidence of acceptable reliability.

**Figure 1: Ab-I_(mod)_: Information across the construct**

Key: Test information curve - solid line; Standard error curve - dotted line.

**Figure 2: Ab-A_(mod)_: Information across the construct**

Key: Test information curve - solid line; Standard error curve - dotted line

**Figure 3: Ab-P_(mod)_: Information across the construct**

Key: Test information curve - solid line; Standard error curve - dotted line.

**Summary**

Statistically separable measures of impairment, activity limitation and participation restriction were established based on items from the Ab-IAP. The separable measures appear to have acceptable validity although further work needs to be done to investigate the psychometric properties of the measures in new samples of participants. It may be important to add items to increase the discrimination of the measures for samples with either very high or low levels of disability. It would also be useful to clarify why measures demonstrating distinct constructs in expert rating studies, did not function independently when completed by patients. It may be that items are contaminated by other constructs, not included in the ICF, resulting in spurious relationships between items. Additionally, patients may make different distinctions from academic experts. It would be useful to have people with clinical conditions affecting functioning to act as expert judges to explore whether they identify similar distinctions to those made by clinical and research experts.

Reference List

1. Pollard B, Dixon D, Dieppe P, Johnston M: **Measuring the ICF components of impairment, activity limitation and participation restriction: an item analysis using classical test theory and item response theory.** *Health Qual Life Outcomes* 2009, **7:** 41.

2. Bentler PM: **Comparative Fit Indexes in Structural Models.** *Psychological Bulletin* 1990, **107:** 238-246.

3. Satorra A, Bentler PM: **Corrections to test statistics and standard errors in covariance structure analysis.** In *Latent variables analysis:Applications for developmental research*. Edited by von Eye A, Clogg CC. Thousand Oaks, CA: Sage; 1994:399-419.

4. Bentler PM: **On the fit of models to covariance and methodology to the *Bulletin*.** *Psychological Bulletin* 1992, **112:** 400-404.

5. Bentler PM, Bonett DG: **Significance tests and goodness of fit in the analysis of covariance-structures.** *Psychological Bulletin* 1980, **88:** 588-606.

6. Browne MW, Cudeck R: **Alternative ways of assessing model fit.** In *Testing structural equation models*. Edited by Bollen KA, Lond JS. Newbury Park, CA: Sage; 1993:136-162.

7. Samejima F: **Estimation of a latent ability using a response pattern of graded scores.** *Psychometric Monograph* 1969, **Supplement No.17**.

8. Ware JE, Sherbourne CD: **The MOS 36-item short form health survey (SF-36) .1. Conceptual framework and item selection.** *Medical Care* 1992, **30:** 473-483.
