# Supplementary material for: Exploring the relationships between International Classification of Functioning, Disability and Health (ICF) constructs of Impairment, Activity Limitation and Participation Restriction in people with osteoarthritis prior to joint replacement
Source: BMC Musculoskelet Disord. 2011 May 16;12:97. doi: 10.1186/1471-2474-12-97 (PMC3123258; doi:10.1186/1471-2474-12-97)
Supplement: Additional file 2 — The measurement model: CFA. Details of the measurement model using CFA including comparisons of the three factor model with alternative one and two factor models. [file 1471-2474-12-97-S2.DOC]

**The measurement model: CFA**

To explore if the three factor model was more appropriate than a one or two factor structure, comparisons with alternative models were made (i.e. one (IAP), two (IA,P; AP,I; IP,A) and three (I, A, P) factor models). Nested models can be compared by calculating the differences in Chi-square as this is also Chi-Square distributed with the degrees of freedom being the difference in degrees of freedom between the two models. Only if the three factor model had significantly better fit than any of the two or the one factor model, would it be considered the most appropriate measurement structure.

The CFA analysis indicated good item fit for the three factor model with correlated factors [SB Chi (149) =439.61; CFI robust=0.91; RMSEA robust= 0.07 (CI 0.06-0.08)]. The other alternative one and two factor models did not have acceptable fit (see Table 1). The three factor model was significantly better than any of the two factor models and significantly and substantially better than the one factor model (see Table 2). In the three factor model all latent constructs were significantly correlated with each other, with the strongest correlations between I and A (*r*=0.76) and A and P (*r*=0.76) and then I and P (*r*=0.58).

**Table 1: Measurement Model: CFA on the modified Ab-I, Ab-A, Ab-P measures**

| **MODEL** | **Chi-sq** | **Df** | **p** | **SB**  **Chi-sq** | **p** | **CFI**  **robust** | **RMSEA**  **robust** | **RMSEA**  **robust CI** |
| --- | --- | --- | --- | --- | --- | --- | --- | --- |
| One factor: IAP | 899.86 | 152 | <0.000005 | 857.79 | <0.000005 | 0.78 | 0.11 | 0.10-0.12 |
| Two factors I-AP with correlated factors | 689.72 | 151 | <0.000005 | 643.05 | <0.000005 | 0.84 | 0.09 | 0.09-0.10 |
| Two factors IA-P with correlated factors | 655.53 | 151 | <0.000005 | 624.95 | <0.000005 | 0.85 | 0.09 | 0.08-0.10 |
| Two factors IP-A with correlated factors | 799.40 | 151 | <0.000005 | 764.60 | <0.000005 | 0.81 | 0.10 | 0.10-0.11 |
| **Three factor I-A-P with correlated factors** | **469.39** | **149** | **<0.000005** | **439.61** | **<0.000005** | **0.91** | **0.07** | **0.06-0.08** |

Key: Chi-sq= Chi-squared statistic, SB Chi= Satorra-Bentler Chi-squared statistic, df=degrees of freedom, CFI robust= robust comparative fit index, RMSEA robust=robust Root Mean Squared Error of Approximation, CI=Confidence Interval.

Bold=acceptable model fit i.e. robust CFI>0.9 and robust RMSEA<0.08.

**Table 2: Measurement Model: Testing the differences between nested models**

| **Model** | **Chi-square difference** | **Df** | **p** |
| --- | --- | --- | --- |
| 3 factor I-A-P v 1 factor IAP | 495.49 | 3 | <0.00005 |
| 3 factor I-A-P v 2 factor I-AP | 253.78 | 2 | <0.00005 |
| 3 factor I-A-P v 2 factor IA-P | 215.49 | 2 | <0.00005 |
| 3 factor I-A-P v 2 factor IP-A | 367.57 | 2 | <0.00005 |

Key: df=degrees of freedom.
